# Minimizing Entropy and Complexity in Creative Production from Emergent Pragmatics to Action Semantics

**DOI:** 10.3390/e26050364

**Published:** 2024-04-26

**Authors:** Stephen Fox

**Affiliations:** VTT Technical Research Centre of Finland, FI-02150 Espoo, Finland; stephen.fox@vtt.fi

**Keywords:** active inference, assembly index, assembly theory, complexity, creativity, entropy, generative, latent, manifest, pragmatics, production, semantics, survival, world models

## Abstract

New insights into intractable industrial challenges can be revealed by framing them in terms of natural science. One intractable industrial challenge is that creative production can be much more financially expensive and time consuming than standardized production. Creative products include a wide range of goods that have one or more original characteristics. The scaling up of creative production is hindered by high financial production costs and long production durations. In this paper, creative production is framed in terms of interactions between entropy and complexity during progressions from emergent pragmatics to action semantics. An analysis of interactions between entropy and complexity is provided that relates established practice in creative production to organizational survival in changing environments. The analysis in this paper is related to assembly theory, which is a recent theoretical development in natural science that addresses how open-ended generation of complex physical objects can emerge from selection in biology. Parallels between assembly practice in industrial production and assembly theory in natural science are explained through constructs that are common to both, such as assembly index. Overall, analyses reported in the paper reveal that interactions between entropy and complexity underlie intractable challenges in creative production, from the production of individual products to the survival of companies.

## 1. Introduction

**Figure 1:**
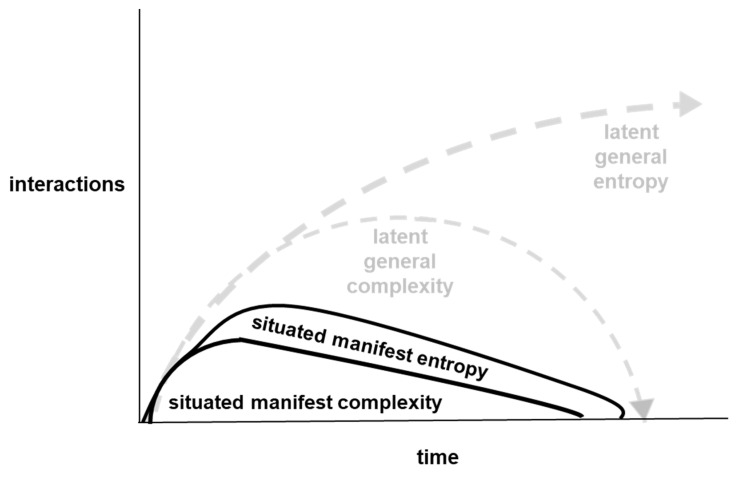
Situated perspective of interactions between entropy and complexity.

It has been argued that “the primordial confrontation underlying the existence of our Universe can be conceived as the battle between entropy and complexity” [1]. The potential for interactions between entropy and complexity is apparent from their generic definitions. In particular, entropy can be described as the number of different ways that a set of objects could be arranged, and complexity can be described as the amount of information needed to describe a system [2]. Interrelationships between entropy and complexity can be illustrated by the mixing of two paint colors to make another paint color. Consider, for example, a container containing blue paint to which yellow paint is added in order to make green paint. The particles in the yellow paint could be arranged in many different ways and the particles in the blue paint could be arranged in many different ways. However, complexity is low because there can be a simple description: blue paint on the bottom and yellow paint on the top. Subsequently, both entropy and complexity increase when the paints are stirred and begin to mix together. This is because there are more ways in which the paint particles could be arranged, and because more information is needed to describe the many color patterns that emerge during the mixing process. Then, when the blue and yellow have been fully mixed together to make green, entropy can still be high because there are many ways in which the particles in the mixed paint could be arranged, but complexity is low because the paint can be described in one word: green. Such interactions between entropy and complexity [3] are summarized by the grey dotted lines in Figure 1 below.

Figure 1 summarizes a situated perspective of interactions between entropy and complexity. As explained in more detail in subsequent sections of the paper, general tendencies for entropy to increase and for complexity to rise then fall can be latent tendencies at a particular place and time. These latent tendencies can be managed, for example, through application of industrial engineering methods, which can lead to the entropy and complexity that manifest in a particular situation to be much lower.

In this paper, interactions between entropy and complexity are analyzed from the perspective of organizational survival, which is dependent upon the production of creative products. Here, the creative product example is bicycles that have personalized storage containers attached to them, i.e., personalized cargo bikes [4]. There are many creative options for one-of-a-kind cargo bikes [5], including shapes and positions of storage containers, patterns and colors of cargo-bike decorations.

Previous studies published in the journal Entropy have related the complexity of products to countries’ economic development [6,7]. Other studies published in the journal Entropy have encompassed creativity. For example, at the macroeconomic level, it has been argued that the tendency towards maximum entropy promotes widespread creativity [8]. At the level of the individual, it has been argued that creativity is a consequence of the human brain’s energy efficiency [9]. However, previous studies have not addressed interactions between entropy and complexity in creative production. This is an important research gap because creative product lifecycles can foster innovation [10] and can provide an important pathway to economic development [11].

In this paper, interactions between entropy and complexity are analyzed in terms of progressions from emergent pragmatics to action semantics during the production of creative products. Within pragmatics, product definitions are based on implicit knowledge of a product’s characteristics. By contrast, within semantics, product definitions are based on explicit representations of a product’s characteristics. In Section 2, the nomenclature used in the paper is explained. The nomenclature distinguishes between general and situated, and between latent and manifest. In Section 3, a practical example of a cargo bike company is described. In Section 4, interactions between situated latent entropy and situated latent complexity are explained. In Section 5, transitions to situated manifest entropy and situated manifest complexity are explained. Broader implications from the analysis are discussed in Section 6. Overall, by distinguishing between latent and manifest, a contribution is made to theory concerned with interactions between entropy and complexity. This contribution is related to assembly theory, which is a recent theoretical development in natural science that addresses how open-ended generation of complex physical objects can emerge from biology [12,13,14].

## 2. Nomenclature

**Figure 2:**
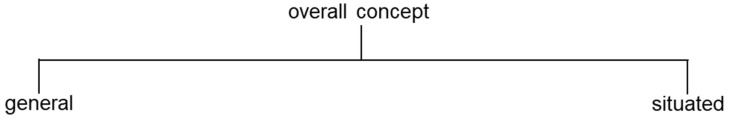
General/Situated.

As summarized in Figure 2, one overall concept can be described in general terms or in situated terms. Examples include situated action, situated knowledge, and situated learning [15,16]. Similarly, pragmatics is concerned with how situations influence communication through the prior knowledge of people and the characteristics of the communication setting [17,18,19]. By contrast, semantic descriptions are general descriptions that can have the same meaning to many people in many settings.

**Figure 3:**
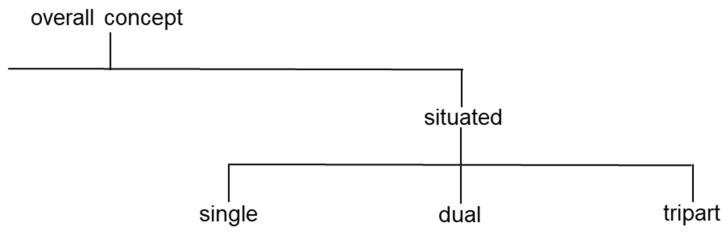
Situated: single, dual, tripart.

As illustrated in Figure 3, an overall concept may have several main elements. For example, switching costs is a conceptualization that is concerned with the costs involved in a person switching from a currently preferred product and/or brand to another product and/or brand. Switching costs can be procedural, financial, and/or relational [20]. In some situations, initially, there may only be the procedural costs of making arrangements, i.e., single. Subsequently, financial costs may also come into effect, i.e., dual, and then relational costs may begin, i.e., tripart.

**Figure 4:**
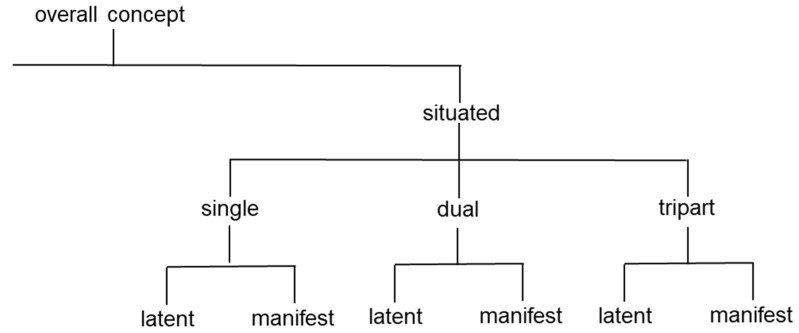
Situated: latent/manifest.

As summarized in Figure 4, the elements of a concept may be latent, or they may manifest in a particular situation. For example, no financial costs may manifest in switching from one product to another product, provided that the customer stays with the current product for a contractually predefined minimum period of time [21]. Distinction between latent and manifest is made in diverse fields such as applied design, conflict management, and entrepreneurship studies [22,23,24].

Entropy can also be considered in terms of latent and manifest. For example, the potential to access a larger number of states is a latent entropy as it refers to the number of states that could possibly be accessed. The states that are accessed are the states that manifest [25]. Also, complexity can be considered in terms of latent and manifest. For example, it has been argued that latent attractors in a complex system may not be visible when one of them is manifest: “However, these latent attractors may be very important in the long run because they determine which states are possible for the system when conditions change” [26]. Distinguishing between latent and manifest can resolve conflicts between positive and negative perspectives [8]. As is explained in more detail in subsequent sections with the practical example, maximizing latent entropy and complexity can enable maximum potential for adaptability within changing environments (positive), but survival may not be possible if manifest entropy and manifest complexity are not minimized (negative).

Entropy and complexity can comprise several elements, which may or may not manifest in a particular situation. For example, there can be information–theoretic entropy, statistical physics entropy, and thermodynamic entropy. Also, complexity can be considered in terms of several elements, which may or may not manifest in a particular situation. For example, creative product lifecycles are characterized by what can be described as dynamic complexity, which combines complicatedness, unpredictability, and frequent changes. Some creative products are more complicated than others. For example, an ocean-going cruise liner has many more components and interconnections between them than a cargo bike. Unpredictability arises from individual customers having authority over production. This leads to each creative product having different initial conditions, which limits the potential for the application of fully predefined specifications for production. Subsequently, frequent changes arise during creative production as iterations between customer and producer lead to the refinement of vague initial product concepts into detailed completed products [27].

In terms of the Ladder of Abstraction, entropy and complexity are high-level concepts. The Ladder of Abstraction provides a structure for the organization of different levels of abstraction from concrete to abstract [28,29,30]. However, both entropy and complexity can become more concrete for practitioners when, rather than being thought of as general concepts, they are related to the particularities of specific situations, such as, for example, the creative production of one-of-a-kind cargo bikes.

## 3. Practical Example

A fictitious company called CBikes is in the cargo bike market. In order to be able to survive and grow in this environment, CBikes needs to adapt with changing market trends. At the same time, CBikes must maintain internal stability by minimizing information uncertainty in its interactions with customers. In particular, information uncertainty that would entail physical disorder and consequent energy loss, which can prevent CBikes from growing or even surviving. In other words, CBikes needs to adapt with its environment while minimizing information–theoretic entropy that could entail statistical physics entropy and consequent thermodynamic entropy [31]. CBikes entered the cargo bike market by offering customers predefined options for cargo bikes, i.e., standardized cargo bikes. As a consequence of limiting its offerings to predefined cargo bikes, CBikes failed to attract many potential customers who want one-of-a-kind cargo bikes. In order to reduce information uncertainty about its survival, CBikes begins to offer one-of-a-kind cargo bikes.

Hence, CBikes must adapt its online configuration platform to be able to facilitate individual customers’ one-of-a-kind orders for cargo bikes, and to facilitate engineer-to-order production (ETO) of such cargo bikes. Here, the term production encompasses design and manufacture. Online configuration platforms are online brochures that can enable users to select and configure sub-assemblies and finishes into their preferred products [32]. The introduction of online configuration platforms is accompanied by extensive industrial engineering, which is carried out to minimize information uncertainty, physical disorder, and energy loss from initial interactions with customers through to product completion. Configuration platforms can enable companies to mediate between adapting with the external market and maintaining internal stability. In the short term, internal stability can be facilitated by not updating the product variety offered via configuration platforms. For example, CBikes could continue to offer only standard cargo bikes. However, not updating product variety can lead to not surviving due to insufficient adaptation with environmental changes such as increasing demand from potential customers for one-of-a-kind cargo bikes.

For companies, information from new external markets begins with emergent pragmatics. Subsequently, it is formalized by companies as semantic information [33]. Pragmatics can encompass physical objects and related descriptors, which can include language and images [34]. As summarized in Equation (1) [35], pragmatics involves Bayesian inference about the state of the world (w). Here, the business’s recipient (_B_) of a customer’s (_C_) signal (s) infers what the state of the world (w) is likely to be now, given that the customer (_C_) produced the signal (s) and knowing that the customer (_C_) is reasoning about how the business’s recipient (_B_) is most likely to interpret that signal (s) based on (w). In other words, without information about the specific context (w), pragmatic information can be ambiguous.

P_B_(w|s) ∝ P_C_(s|w)P(w) (1)

By contrast, within semantics, information is predefined, standardized, and explicit. Semantic information can encompass physical objects and the actions related to them, i.e., action semantics [36]. The semantics used in describing creative products and the work involved in their production can include natural language, machine-readable language, visual images, and physical objects [32,33].

When the founders of CBikes were exploring the possibility to set up a cargo bike company, they may have had discussions that were entirely dependent on pragmatics because there may have been no standard sub-assemblies and associated descriptors for cargo bikes. Rather, cargo bikes may have been the individual fabrications of ingenious hobbyists [4,5]. Subsequently, when developing a configuration platform for predefined options for cargo bikes, they would have standardized sub-assemblies and the associated descriptors. Then, the CBikes configuration platform, as with other configuration platforms, would be based on semantic information [33]. This is information that can have the same meaning for customers outside the company and for all those within the company. Semantic information can be considered to be disambiguated pragmatic information that is generalizable beyond a few specific situations and specific people.

Production information can be considered to be semantic information when it has zero information–theoretic entropy because it can only be interpreted in one way. When there is zero information–theoretic entropy, production work can be carried out correctly first time every time. By diversifying its market offerings to encompass individual customers’ requirements for one-of-a-kind cargo bikes, CBikes is opening its production processes to pragmatic information. This is because the informal descriptions of requirements provided by each individual customer are founded upon those customers’ personal knowledge and particular situations.

Thus, paradoxically, in trying to reduce information uncertainty about its survival, which has been caused by insufficient sales of standard cargo bikes, CBikes has to risk undermining its survival by being open to high information–theoretic entropy from the pragmatics of individual customers’ ambiguous information about one-of-a-kind cargo bikes. This can undermine CBikes’ survival because it entails increased information uncertainty, which can permeate throughout production processes to bring physical disorder and consequent energy loss. This is a practical example of the fundamental need to balance external adaptability and internal stability in order to survive and grow in changing environments [31].

## 4. Interactions between Latent Entropy and Latent Complexity

**Figure 5:**
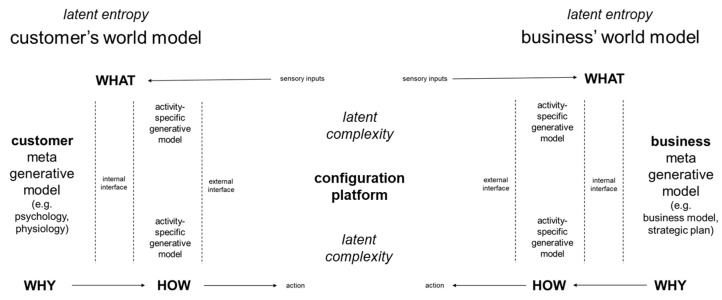
World models comprising generative meta models and activity-specific models.

Natural science’s assembly theory is concerned with the open-ended generation of novelty through a forward dynamical assembly process [14]. As summarized in Figure 5, for practical purposes, interactions between latent entropy and latent complexity can be framed in terms of generative world models and the interface states between them.

World models are models of self in the world, which can generate cycles of inferences about survival in changing environments. As shown in Figure 5, a world model can comprise one meta model that influences many activity-specific models. A meta generative model can encompass characteristics that influence many different activities in many different situations and provide the basis for why actions are taken in the world. For a business, its meta model can be defined within documents such as the description of its business model within its strategic plan documents. Activity-specific generative models provide the basis for how actions are taken in the world. For example, generative models for production activities, which can be documented as work procedures within the business’s quality management system manuals and within related computational models [37]. These activity-specific models influence what sensory inputs are experienced from actions taken in the world. By contrast, the world models of individual people are embodied psychomotor world models [38]. For individual customers, meta generative models can comprise underlying psychological and physiological characteristics, such as personality type and soma type, which can influence a person’s inferences in all the many different types of activities that they perform. For example, a person who has an inventive personality type [39] and a mesomorph soma type [40] might want to have a one-of-a-kind cargo bike to transport heavy goods by peddle power.

As explained in Section 2, the potential to access a larger number of states is a latent entropy, as it refers to the number of states that could possibly be accessed [25]. When CBikes decided to offer to produce one-of-a-kind cargo bikes, CBikes updated its meta model from being a business that made standard cargo bikes, i.e., a make-to-stock (MTS) business, to being an engineer-to-order (ETO) business. This update entailed the updating of activity-specific models for production, which requires an increase in latent entropy as CBikes needs to increase potential for adaptive behavior in its operations to be able to deal with the unpredictability and changing characteristics of customer requirements for one-of-a-kind cargo bikes. As discussed in Section 2, creative product lifecycles are characterized by what can be described as dynamic complexity, which combines complicatedness, unpredictability, and frequent changes [27]. A measure of the complexity [2] of a creative product can be the amount of information needed to describe it at any specific time, with more information being needed to describe higher dynamic complexity than to describe lower dynamic complexity. Before they begin to be described explicitly in product-specific sketch drawings, etc., the complexity of creative products is a latent complexity, which can be framed as being a latent state space. That is the set of all possible states of the dynamical system [41] for the creation of one-of-a-kind cargo bikes. Variables of the latent state space include, for example, the possible shapes and possible decorations of cargo bike storage containers. The specific values of all variables in any one case cannot be predicted fully in advance [42]. However, a configuration platform can be set up to establish a latent solution space for the production of cargo bikes. The term solution space, which may also be referred to as feasible region, can provide a framework for constraint satisfaction optimization. In constraint satisfaction problems, a solution can be defined in terms of a set of constraints that impose conditions that variables must satisfy [43,44]. In this case, there can be regulatory constraints and customer constraints for cargo bikes, which need to be satisfied by variables such as the shapes and the decorations of cargo bike storage containers.

Interactions between latent entropy and latent complexity in creative production arise from it not being possible to define values for all variables in advance. Regulatory constraints can be defined in advance, but customer constraints cannot be defined in advance. For example, minimum and maximum sizes for cargo bike wheels can be defined in advance as minimum and maximum variable values that satisfy regulatory constraints for the roadworthiness of cargo bikes. However, it cannot be predicted in advance what what shape and what decorations any individual customer will imagine for a cargo bike’s storage container, and how much a customer will be prepared to pay for a unique cargo bike. Rather, customer constraints can only be defined as follows: if the customer is satisfied, then the customer will pay and CBikes can survive. Thus, CBikes’ openness to allowing individual customers to have authority over the production of cargo bikes increases the number of different ways in which the set of variables in cargo bike production could be arranged; i.e., latent entropy increases. Hence, the amount of information that could be needed to describe all possible cargo bikes increases; i.e., latent complexity increases.

## 5. Transitions to Manifest Entropy and Manifest Complexity

### 5.1. Transition Process

**Figure 6:**
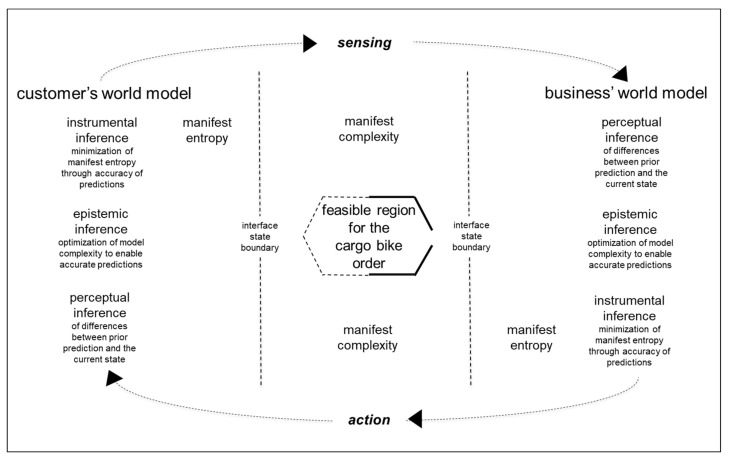
Generative iterations of active inference from emerging pragmatics to action semantics.

Natural science’s assembly theory is concerned with open-ended generation of novelty through a forward dynamical assembly process [14]. As summarized in Figure 6, definition of the feasible region for each individual cargo bike order involves Bayesian iterations of active inference between the customer and the business: i.e., iterations of perceptual, epistemic, and instrumental inference. These iterations of active inference are generated by the customer’s and the business’s world models.

Perceptual inference involves inferring external sensory stimuli from predictions based on internal representations that are within world models. Epistemic inference includes updating internal representations in world models related to survival in an environment. Instrumental inference involves inferring action options and their consequences for survival in the environment. Perceptual inference is important for survival because it enables perception of differences between prior predictions about action consequences and the current state of the environment, i.e., perception of prediction errors that can undermine survival. Epistemic inference is important for survival as it enables updating of world models to match the complexity of the environment in which survival is intended. Epistemic inference can entail learning about the environment, which updates world model structure (e.g., to include new types of cargo bikes), and/or world model parameters (e.g., cargo bikes can be between one meter and two meters long). Optimal fit of the world model with the environment is necessary to enable planning of actions through instrumental inference, which can be predicted accurately to enable survival in the environment through minimization of manifest entropy. Such iterations of active inference are necessary for optimal fit of a meta model and for optimal fit of activity-specific models. For CBikes, its activity-specific models encompass production activities such as the definition of feasible regions, i.e., solution spaces, for individual customers’ cargo bike orders.

About half of the feasible region shown in Figure 6 is already predefined (solid lines), because regulatory requirements for cargo bikes are already well-defined. However, the definition of customer-specific requirements is open (dotted lines). Active inference iterations for the definition of the open feasible region for a cargo bike order begin with a nascent idea for a new cargo bike in the mind of a customer. As summarized in Equation (1) [35], pragmatics involves Bayesian inference about the state of the world (w). As summarized in Equation (2), for practical purposes in the production of creative products, the state of the world can be reduced to the state of the feasible region (fr) for that product. Here, CBike’s recipient (_B_) of the customer’s (_C_) signal (s) infers what the state of the feasible region (fr) is likely to be now, given that the customer (_C_) produced the signal (s) and knowing that the customer (_C_) is reasoning about how the business’s recipient (_B_) is most likely to interpret that signal (s) based on (fr).

P_B_(fr|s) ∝ P_C_(s|fr)P(fr) (2)

Within engineering design, active inference iterations about a feasible region (fr) can be made explicit by iterations of rough sketch drawings, which initially need only be sufficiently detailed to facilitate shared information uncertainty reduction between _B_ and _C_. Such sketches can provide visual summaries of the implicit shared knowledge being developed between _B_ and _C_ during iterations of active inference. Thus, sketch drawings can provide visual pragmatics as they emerge between _B_ and _C_. However, in order to minimize manifest entropy, these visual pragmatics need to be converted into action semantics [36] before any production actions are taken by CBikes.

Within natural science’s assembly theory, it is argued that “The more complex a given object, the less likely an identical copy can exist without selection of some information-driven mechanism that generates that object” [14]. This argument has been formulated to differentiate between objects that are generated through a selection mechanism for reproduction and objects that are generated in series of random events. Here, an identical copy does not refer to the physical details of one-of-a-kind cargo bikes, which are intentionally different, but to the economic characteristics of a one-of-a-kind cargo bike being bought by an individual customer for a price with a profit margin that enables CBikes to survive and grow. In this case, the information-driven mechanism that generates cargo bikes is exchanges between CBikes and its customers that are facilitated by CBikes’ online configuration platform. The exchanges between CBikes and its customers are generated by their world models through iterations of active inference.

### 5.2. Peak Manifest Entropy and Peak Manifest Complexity

**Figure 7:**
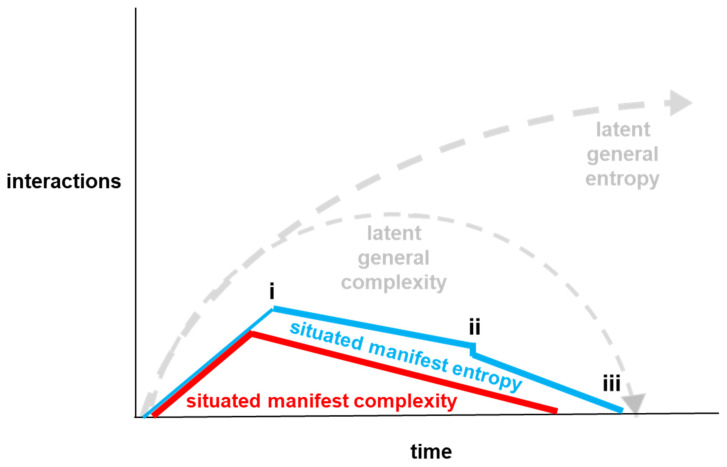
Interactions between situated entropy and situated complexity: (i) transition from manifest information–theoretic entropy in design work (thin blue line) to manifest tripartite entropy in manufacturing work (thicker blue line) begins after the amount of information needed to describe cargo bike manufacturing has begun to decline because of its definition in multimodal semantics (red line); (ii) there is a spike in manifest tripartite entropy (thicker blue line) at place and time where fiberglass storage container is fitted to cargo bike steel frame while the amount of information needed to describe the cargo bike decreases (red line); (iii) both manifest entropy (blue line) and manifest complexity (red line) are minimal when the cargo bike is completed and can be observed easily.

As shown in Figure 7, at point i, the amount of information needed to describe the cargo bike can rise to a peak (red line) before every detail of the cargo bike’s production is defined in multimodal semantics.

Manifest information–theoretic entropy (thin blue line) should peak before manufacturing work begins. This is because manifest information–theoretic entropy in manufacturing work can lead to manifest physical statistics entropy that entails manifest thermodynamic entropy in what can be described as situated manifest tripartite entropy (thicker blue line). As discussed in Section 2, the term situated manifest tripartite entropy can be used to describe the combination of information–theoretic entropy, statistical physics entropy, and thermodynamic entropy at a particular place and time. For practical purposes, tripartite entropy can be described as combinations of information uncertainty, physical disorder, and energy loss. The concluding sketch-drawing pragmatics between CBikes’ salesperson _B_ and the customer _C_ may entail little, if any, information uncertainty for them, but can entail high information uncertainty for anybody else. Hence, there can be high physical disorder and energy loss for CBikes if physical work begins before there is information certainty for all variable values in the feasible region, i.e., the solution space, for this customer’s order. For example, there can be information uncertainty of 2.58 bits if the assembly instructions for the cargo bike can be interpreted in six different ways with equal probability. If only one of these six ways is the correct way to assemble the cargo bike, there will be physical disorder in five out of six attempts. In particular, there is the physical disorder of assembling the cargo bike incorrectly, then disassembling the cargo bike, followed by trying again to assemble it correctly. This physical disorder entails irreversible energy loss through unproductive energy expenditure. In this example, tripartite entropy arises from there being six different ways in which the cargo bike can be assembled. This corresponds with the generic definition description of entropy as “a measure of the number of different ways a set of objects can be arranged” [2,45]. This example illustrates that, although experiences of situated manifest entropy can be subjective, computation of situated manifest entropy can be observer-independent and can enable different observers, such as factory supervisors and business managers, to have access to the same information. Detailed examples of situated entropy calculations can be found in [46].

Industrial engineering methodologies can be applied to prevent situated manifest tripartite entropy without increasing manifest complexity in production work. In other words, reduce the number of ways a product can be assembled to one without increasing the amount of information needed to describe the assembly work. For example, the industrial engineering methodology, Design for Assembly (DFA) is applied to facilitate assembly work being done correctly the first time by any production worker. An example of this is consolidating many simple small parts, such as metal sections, bolts, nuts, washers, into a few larger components, which can only be put together in one way. Parts consolidation can reduce the DFA assembly index for a product, which incorporates definition of minimum number of parts and minimum assembly time per part [47,48]. Interestingly, the construct of assembly index is also central to natural science’s assembly theory that addresses how open-ended generation of complex physical objects can emerge from selection. Within assembly theory, “the assembly index of an object is the length of the shortest pathway to construct the object starting from its basic building blocks” [13].

### 5.3. Small Spike in Manifest Entropy despite Decreasing Manifest Complexity

The example of there being six different ways to assemble a cargo bike is situated tripartite entropy because the entropy relates to information uncertainty, physical disorder, and energy loss in a particular situation: the assembly of a cargo bike at a specific place and time. However, it is important to distinguish between situated latent tripartite entropy and situated manifest tripartite entropy. The entropy is latent before the assembly work begins. If the assembly work is never done, then that situated latent tripartite entropy may never manifest. If the assembly work is done, there can be different amounts of situated manifest tripartite entropy. This is because the extent to which situated tripartite entropy manifests is determined by situation-specific variables. In this example, situation-specific variables could be the level of assembly experience of two different assembly workers. A novice could have five unsuccessful assembly attempts on average, but an expert might draw upon past experience and need only one attempt to assemble the cargo bike correctly. Thus, it is not inevitable that all situated latent tripartite entropy becomes situated manifest tripartite entropy.

Industrial engineering seeks to turn the implicit knowledge of individual expert production workers, which can be exchanged among expert production workers through pragmatics, into action semantics that can be actioned correctly first time by novice production workers. This can be facilitated through action semantics [36] in the form of standardized production tools that are developed for specific production operations, such as specific assembly tasks. For example, physical tools called jigs can be used to facilitate positioning, supporting, and fixing components during assembly work [49]. Nonetheless, as shown in Figure 7, at point ii, there can be a spike in situated manifest tripartite entropy when the irregular curved shape of a one-of-a-kind fiberglass storage container is fitted to the regular straight shape of a cargo bike’s steel frame. This is because it is not economically viable to manufacture individual jigs for one-of-a-kind storage containers. Rather, there has to be some trial and error involved in the fixing work, which can lead to a small spike in manifest entropy. However, this does not entail a spike in manifest complexity, as there is no need for increased information to describe the production work. This is because compatible tolerances can be defined for the manufacture of glass fiber storage containers and the fixing of them to cargo bike steel frames [50]. For example, the diameter of a curved storage container can be plus/minus 10 mm, and the overall dimensions of the complete cargo bike can also be plus/minus 10 mm. Furthermore, there can be a corresponding tolerance in the time schedule for the assembly work, which can be described as slack [51]. In terms of natural science’s assembly theory, the formulation of jigs and tolerances can be considered to be in terms of memory within an assembly space that enables the minimal number of operations necessary to construct an object [14].

### 5.4. Minimal Manifest Entropy and Minimal Manifest Complexity

As shown in Figure 7, at point iii, both manifest entropy (blue line) and manifest complexity (red line) are minimal when production of the cargo bike has been completed. There is minimal manifest entropy because the measure of the number of different ways a set of objects can be arranged is zero, as the set of objects (i.e., cargo bike components) have all already been arranged and fixed together. Manifest complexity is minimal because the amount of information needed to describe the cargo bike is greatly reduced, as it is now one completed product in one place rather than many components in many places.

After each one-of-a-kind cargo bike has been completed, CBikes can perform iterations of active inference with the aim of trying to reduce the peak of manifest entropy and the peak of manifest complexity for future orders. This can involve epistemic inference to update its activity-specific model for production with multimodal semantic descriptions of cargo bike components that could be used again in future orders. These descriptions could be in the form of digital component descriptions that can include digital images that can be viewed by customers and related digital component descriptors that can be used by CBikes for production. The use of such digital component descriptions in CBikes’ configuration platform can facilitate perceptual inferences that are shared by CBikes and its customers. Shared perceptual inference can be followed by shared instrumental inference about which actions should be taken in the physical production of the cargo bike. For example, there could be shared instrumental inference that the storage container should be positioned in front of the person peddling rather than positioned behind the person peddling. In terms of natural science’s assembly theory, the repeated use of the same components in future objects can be considered in terms of copy number. For example, “once the pathway for a new object has been discovered, the production of an object gets easier as the copy number increases because a high copy number implies that an object can be produced readily in a given context” [14]. These words from natural science’s assembly theory provide a summary of everyday practice in industrial engineering within human organizations’ quality management systems, which are intended to make production increasingly easy over time.

However, it is important to recognize that the reduction of situated manifest entropy and situated manifest complexity by one business through an online configuration platform and associated industrial engineering does not reduce them universally. Rather, reducing them locally can lead to them increasing elsewhere. For example, more digital component descriptions for an online configuration platform depends upon there being more computer hardware, which depends upon more mining for rare earth metals, more intercontinental transportation of metals, more energy intensive metals processing, etc.: all of which can increase manifest entropy and manifest complexity in accordance with the representation of general entropy and general complexity [3] illustrated in Figure 1.

## 6. Discussion

Analyses of interactions between entropy and complexity can provide insights into interactions between physics and biology [12,13,14]. For example, the aim of natural science’s assembly theory is “to develop a new understanding of the evolution of complex matter that naturally accounts for selection and history in terms of what operations are physically possible in constructing an object” [14]. In this paper, the “complex matter” includes cargo bike components and production tools. “Selection” of “physically possible” “operations” “in constructing an object” of a cargo bike arises from the need to survive in the changing environment of cargo bike markets. In particular, CBikes needed to “select” operations that are “physically possible” for “constructing” one-of-a-kind cargo bikes. CBikes needed to do this because it could not achieve enough sales to survive by offering standard cargo bikes. Components and tools for one-of-a-kind cargo bikes have a different “history” than physical “operations” that are “physically possible” for “constructing” standard cargo bikes.

Companies’ “selection” of operations with a fitting “history” in order to survive in changing environments can be motivated by wanting to avoid the internal shame and external stigma that can be associated with financial bankruptcy [52]. Here, fitting “history” refers to ecological fitness. For example, the “history” of production tools for mass production (i.e., MTS) of curved containers is a history of increasing investment in component-specific molds and presses, which can produce thousands of components that are exactly the same. By contrast, the “history” of production tools for one-of-a-kind production (i.e., ETO) of curved containers is a history of continuing investment in tools for cutting sheet materials and bending them into unique shapes. CBikes cannot invest in molds and presses for mass production (i.e., MTS) because they do not fit the market environment in which CBikes aims to survive and grow (i.e., ETO). CBikes will not invest in production tools that are not fitting with its environment because of fear of business failure, which can have many grave implications [53,54,55]. More generally, humans are driven to take actions to mitigate threats to survival. Business decision-makers can find themselves impelled by biology [56] and compelled by culture [57] to take new actions in order to survive [58]. Thus, selection for survival is as relevant to industrial assembly practice as it is to natural science’s assembly theory. Moreover, further analysis of industrial assembly practices may provide some insights that could inform further development of natural science’s assembly theory.

Here, industrial production operations have been analyzed in terms of interrelationships between entropy and complexity. This analysis has distinguished between general and situated, and between latent and manifest (Figure 1, Figure 2, Figure 3, Figure 4 and Figure 7). It can be argued that high-situated latent entropy and high-situated latent complexity are both good for business survival. For example, a business with a world model characterized by high latent entropy has high potential to be able to adapt with changing environments (Figure 5). Furthermore, a business with an online configuration platform that is open to defining feasible regions for one-of-a-kind products has high latent complexity, which can attract a wide diversity of potential customers. However, it can be argued that high situated manifest entropy and high situated manifest complexity are both bad for business survival. Hence, industrial engineering is widely deployed in conjunction with configuration platforms (Figure 6). For example, when physical goods, such as cargo bikes, are fully predefined, i.e., standardized, industrial engineering methods such as parts consolidation can be applied fully to reduce situated manifest entropy and reduce situated manifest complexity. In other words, methods such as parts consolidation can reduce the number of ways in which a product can be assembled without increasing the amount of information needed to describe the assembly work. Even when physical goods are not fully predefined, industrial engineering tools, such as jigs, can be used to reduce situated manifest entropy and reduce situated manifest complexity. Overall, configuration platforms and associated industrial engineering can minimize entropy and complexity in creative production from emergent pragmatics to action semantics. One direction for future research in natural science could be to investigate whether this, and other insights from analysis of industrial assembly practice, can be found in microscopic assembly that is driven concurrently by physics and biology.

## Data Availability

No data was used in the research reported in this paper.
